# Bacterial Cellulose Production in Dried Apricot Extract Medium: Experimental/Theoretical Characterization and Application in Yeast Immobilization for Dye Removal

**DOI:** 10.1021/acsomega.5c01126

**Published:** 2025-08-22

**Authors:** Filiz Boran, Suleyman Koytepe, Gizem Aslan, Emre Birhanli, Ozfer Yesilada, Mehmet Abdullah Alagoz

**Affiliations:** † Department of Biology, Arts and Science Faculty, 37520 Inonu University, 44280 Malatya, Turkey; ‡ Department of Chemistry, Arts and Science Faculty, Inonu University, 44280 Malatya, Turkey; § R&D Production Department in Pharmaceutical Industry, Faculty of Pharmacy, Inonu University, 44280 Malatya, Turkey

## Abstract

Bacterial cellulose (BC) was produced in dried apricot extract medium (DAEM) by *Gluconacetobacter xylinus* *B759*. The BC yield obtained from DAEM containing 0.5 g of glucose after 10 days of incubation at 30 °C was determined as 9.67 g/L. BC was used as an immobilization matrix for *Saccharomyces cerevisiae*. First, structural characterizations of BC and BC-yeast were carried out. Also, their surface and morphological properties were examined by SEM and atomic force microscopy. Yeast attachment and growth kinetics were also evaluated experimentally and theoretically. This yeast-immobilized BC (BC-yeast) membrane was used for removal of textile dye, Reactive Blue 171 (RB 171). The results showed that yeast was successfully immobilized on BC and could be effectively used for dye removal. When 12 lyophilized BC-yeast samples were used, 52% and 21% dye removal values were obtained under static and shaking (150 rpm) conditions, respectively. These values were 49% and 50% for wet BC-yeast samples after 24 h, respectively. Additionally, density functional theory calculations were performed to elucidate the interactions responsible for the adsorption of RB 171 dye onto the BC structure, and it was shown that the RB 171 structure is energetically very strong and favorably bound to the BC surface. It was found that BC-yeast was highly effective for RB 171 removal. Therefore, the BC produced in DAEM could be used as an immobilization matrix and as a low-cost, effective, and alternative adsorbent for the removal of dyes from dye-containing wastewaters.

## Graphical abstract

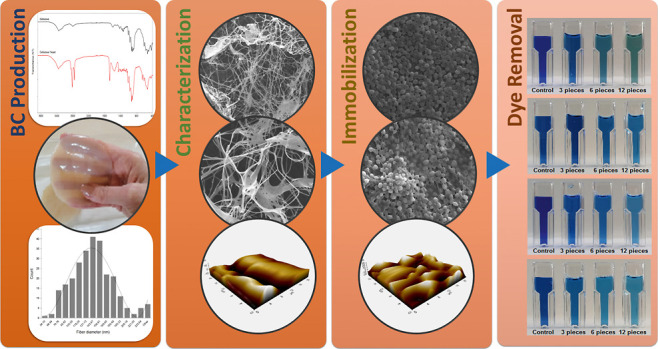

## 1 Introduction

Cellulose is the most common natural polymer and the main component of the basic cell wall structure of many plants. It can be obtained from plant sources, such as wood pulp, cotton, hemp, flax, jute, and ramie fiber. In addition to plants, cellulose is also found in some microorganisms such as bacteria, fungi, and algae.^1–3^ Various bacteria from various genera, such as *Acetobacter*, *Rhizobium*, *Agrobacterium*, *Aerobacter*, *Achromobacter*, and *Zeobacter,* and also some algae produce cellulose as part of their metabolic processes.^4,5^

In 1886, A.J. Brown was first reported that *Acetobacter xylinum* could produce BC.^3,6^ Since its discovery, BC has shown fascinating potential as a sustainable polymer that has attracted a lot of attention in many fields.^7^

Compared to plant cellulose, BC has important and superior properties such as high purity, high mechanical strength, high crystallinity, high liquid loading capacity, biocompatibility, nontoxicity, and biodegradability.^8,9^ All these superior features of BC make it attractive in many industrial and medical fields, such as wound dressing materials, BC membranes, artificial skin, food applications, paper, electrical, and magnetic processes.^8,10^ One of these applications is using BC as a bioadsorbent for the decolorization of textile industry wastewater. Cellulose-based materials show high affinity for certain contaminants such as dyes, and thanks to the chemical groups on its surface, BC helps the adsorption of charged pollutants.^11,12^ Heavy metals such as cadmium and chromium are also removed from the aquatic environments, and environmental pollution can be eliminated by BC.^13^ Therefore, BC has significant potential for removing pollutants from wastewater and polluted water.

A Gram-negative and aerobic bacterium, *Komagataeibacter xylinus* (formerly known as *A. xylinum* and *Gluconoacetobaceter xylinus*), produces a large amount of BC.^4,14^ This is the most commonly used microorganism for BC production. In this study, the BC production potential of *G. xylinus* B759 was evaluated in dried apricot extract media (DAEM) under optimum conditions. BC is also a good naturel polymer for immobilization of microorganisms and enzymes. Therefore, here, the obtained BC was used as an immobilization matrix for yeast and chemical analyses of yeast-immobilized BC (BC-yeast), and BC without yeast cells was also carried out. The dye removal potential of the BC-yeast was also tested.

BC production in cheap and natural media is very important for large-scale cellulose production.^15^ Apricot (*Prunus armeniaca L.*) is a tree belonging to the Rosales group. In addition to having an attractive color and a typical taste, it is a rich source of beta-carotene, ascorbic acid, carbohydrates, vitamins, minerals, and fiber.^16,17^ Especially, dried apricot is rich in vitamins A and C, potassium, iron, folic acid, antioxidants, and fiber. It also contains plenty of sugar in their structure. Therefore, the dried apricot extract was used as a growth medium of *G. xylinus*. During dried apricot production, stained, injured, and spoiled apricots are separated as waste, and these waste apricots constitute a very suitable source for preparing the growth medium of *G. xylinus* for BC production.

To the best our knowledge, this is the first study on BC production using dried apricot extract as a growth medium for microorganisms, and there are limited studies on dye removal abilities in BC-yeast samples. This study, which includes environmental and biotechnological approaches, is important both for the use of medium prepared from a waste raw material in BC production and for dye removal from wastewaters such as textile industry wastewater and, therefore, protection of the environment.

In this study, a high amount of BC was produced with *G. xylinus* B759 using DAEM. Yeast immobilization was carried out after obtaining the fibers. The obtained BC structure was examined in detail by instrumental analysis techniques such as Fourier transform infrared (FTIR) and X-ray diffraction (XRD) spectrum. In addition, the surface morphologies of the produced BC structures were examined by scanning electron microscopy (SEM) and atomic force microscopy (AFM) techniques. The obtained BC fiber structure was found to be quite ideal for yeast immobilization in terms of surface area, morphology, and chemical properties of the surface. The obtained BC-yeast showed quite successful results in terms of dye removal. These results were confirmed by theoretical calculations with DFT analysis. The adhesion mechanism of Reactive Blue 171 (RB 171) dye to BC structure was investigated by computational calculations using DFT/B3LYP-D3/6-31G**/Solv basis sets. Thus, the adhesion activity of the RB171 structure to the BC structure was investigated. In addition, a molecular docking model was used to show the interaction and binding energy between these two molecules, and the obtained results were compared with the experimental results.

## 2 Materials and Methods

### 2.1 Bacterial Strain and Chemicals

The bacterial strain *G. xylinus* B759 was used as the BC producer in the study. Glucose, sucrose, fructose, citric acid, peptone, and media used in the study were obtained from Sigma-Aldrich Company. HCl, NaOH, Na_2_HPO_4_, and other buffer solutions were purchased from Merck. Hestrin-Schramm (HS) agar plates containing (g/L) glucose, 20; peptone, 5; yeast extract, 5; Na_2_HPO_4_, 2,7; citric acid, 1,15; and agar, were used for growing this bacterium. All plates were incubated at 30 °C for 10 days statically and then stored at 4 °C in the refrigerator. It was subcultured monthly.

### 2.2 Preparation of Inoculum

HS broth (consisted of (g/L) glucose, 20; peptone, 5; yeast extract, 5; Na_2_HPO_4_, 2,7; and citric acid, 1,15) was used as the *G. xylinus* growth medium for preparation of stock inoculum culture.^18^ A quantity of solid *G. xylinus* culture was inoculated into a 50 mL volume of HS broth, and the culture was incubated at 30 °C under static conditions for 10 days. Then, 1 mL of this culture was transferred to fresh HS broth and it was statically incubated at 30 °C for 10 days. This was used as the *G. xylinus* stock inoculum culture for BC production studies.

### 2.3 Culture Medium for BC Production

For BC production, a dried apricot extract was chosen as the main culture medium. Dried apricots used to prepare the extract in this study were purchased from the local market. First, 80 g of dried apricots was boiled in 1000 mL of distilled water for 10 min and then filtered. The obtained filtrate (40 mL) and 0.5 g of glucose were added into 250 mL flasks, and the culture media were autoclaved at 121 °C and 1.5 atm. for 20 min. Finally, the culture media were inoculated with 1 mL of stock inoculum culture, prepared as stated above, and they were statically incubated at 30 °C for 10 days. Since the optimum growth temperature for the production of the highest amount of BC was reported as 30 °C in our previous study, this temperature value was used for the growth temperature of this bacterium.^19^

### 2.4 Obtaining of Pure Bacterial Cellulose

After 10 days of incubation, the formed BC sheets were filtered and washed several times with distilled water. BC sheets were then treated with 0.1 M NaOH at 75 °C for 2 h to remove bacteria from cellulose. They were washed with distilled water until neutralization. After this process, wet BC sheets were obtained. For obtaining dry BC sheets, they were lyophilized for 16 h. All experiments were performed in triplicate. After lyophilization, the dry weight of BC was determined as 9.67 ± 0.07 g/L. Figure 1 shows the obtained wet BC sheets before (a) and after (b) neutralization.

**Figure 1:**
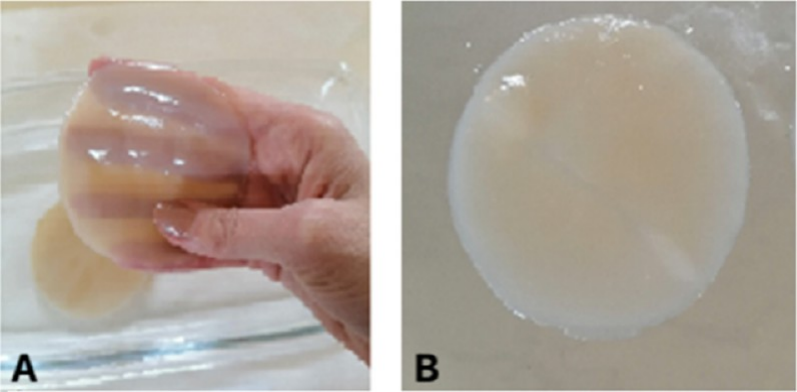
Macroscopic images of wet BC sheets before (A) and after (B) neutralization.

### 2.5 Immobilization of *Saccharomyces cerevisiae* on BC

The produced wet and lyophilized BC sheets were cut into 0.5 cm dimensions, and these pieces were autoclaved in 50 mL of SDB medium (121 °C, 20 min). For preparing the stock culture of *S. cerevisiae*, an amount of solid *S. cerevisiae* was inoculated into 50 mL sterile Sabouraud Dextrose broth (SDB) medium, and this culture was incubated at 30 °C and 150 rpm for 48 h. One milliliter from the stock yeast culture was inoculated into BC-containing media, and all cultures were incubated statically at 30 °C for 48 h for the immobilization. Then, these BC-yeast samples were used for characterization and dye removal studies.

### 2.6 Equipment

The BC-yeast and BC samples were characterized by spectroscopic and microscopic techniques. Then, the surface hydrophilic character of these samples was investigated by liquid contact angle measurements. Initial identification of BC-yeast and BC samples was determined by simple light microscopy (Leica microscope). Then, these samples were characterized using FTIR and XRD spectrophotometers. Chemical characterizations of the produced BC structures were carried out using a PerkinElmer spectrum two model FTIR spectrophotometer. FTIR analyses of these samples were scanned with a working sensitivity of 4 cm^–1^ in the range of 400–4000 cm^–1^. ATR mode was used as the operating mode. The crystallinity level of the BC samples was determined by X-ray analyses using a Rigaku brand X-ray spectrophotometer. The BC samples were measured in the range 2–80° θ, with a measurement sensitivity of 0.2° θ.

Surface and morphological analyses of BC and BC-yeast structures were performed by SEM and AFM analyses. In AFM analysis, 40 μm × 40 μm scanning area was performed using noncontact mode. AFM samples were monitored at room temperature and in Faraday cages using the Park system XE-100 AFM device. Leo-Evo 40 model scanning electron microscope was used for SEM analysis. Before the samples were analyzed, they were coated with a Baltec brand sputter with a 20 m Au/Pd layer.

Shimadzu 1600 UV–vis spectrophotometer was used to determine the absorbance of the dye-containing solutions.

### 2.7 Dye Removal with BC-Yeast Sheets

The dye removal ability of BC-yeast sheets was tested using a textile dye, RB 171. The tested dye concentration was 200 mg/L. 3, 6, and 12 pieces of wet and lyophilized BC-yeast sheets were separately placed into 12 well plates, and then 3 mL of dye solution was added onto them. All plates were incubated at 30 °C for 24 h under static and agitated (150 rpm) conditions. Dye removal was determined by measuring the absorbance changes at the maximum absorbance wavelength (616 nm) of RB 171.

All experiments were performed in triplicate. Dye removal percentages were calculated with the SPSS 15.0 package program.

### 2.8 Theoretical Calculations and DFT Analysis of the Adsorption Mechanism between BC and Reactive Blue 171

The interaction energy and molecular affinity between the synthesized BC and RB 171 were determined by theoretical density functional theory (DFT) calculations with the (Schrodinger) jaguar program. In these calculations, the ground state geometries of BC and RB 171 were optimized by studying them. In these calculations, the B3LYP-D3 (Becke’s three-parameter hybrid functional using the BLYP correlation functional) functional with 631 G**/Solv basis set in aqueous media was used. Relative stability analysis based on binding or adsorption energy (Eads) was performed on the interactions between the two molecules. Adsorption energy was calculated using the following.

$$E_{ads}=E_{complex}-\left(E_{RB-171}+E_{BC}\right)$$

Highest occupied molecular orbital (HOMO), lowest occupied molecular orbital (LUMO), and energy gap (Eg) were also calculated for the complex structure with BC and RB 171. Additionally, electronegativity (χ), dipole moment (μ), and hardness (η) values were also calculated to investigate the chemical stability and suitable interaction points between BC and RB 171.^20–22^

## 3 Results and Discussion

### 3.1 Structural Characterization of BC and BC-Yeast

IR spectra of BC and BC-yeast samples are given in Figure 2. When Figure 2 was examined, –OH stretching vibrations originating from glucose units are clearly seen between 3000 and 3630 cm^–1^. Stretching vibrations of aliphatic C–H groups were also observed between 2830 and 2980 cm^–1^. In addition to these peaks, C–O–C etheric stretching vibrations were observed in the range of 1000–1100 cm^–1^. These vibrations are due to beta-1,4-glucosidic bonds between alhydroglucoses. Especially at values of 1560 and 1314 cm^–1^, there were C–C aliphatic stretching vibrations originating from d-glucopyranose units. In addition, there were C–H symmetrical expansion and out-of-plane stress peaks at the values of 558 cm^–1^ and 660 cm^–1^ depending on these units. There was a C–H rocking peak at 435 cm^–1^. All of these peaks showed that the desired BC structure was formed.

**Figure 2:**
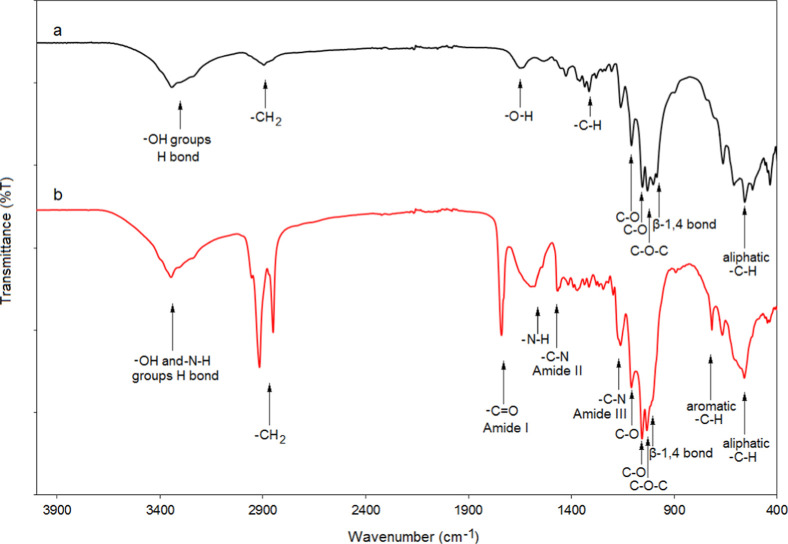
FTIR spectra of BC (a) and BC-yeast (b) samples.

When the IR spectrum of BC-yeast was examined, some significant changes compared to the IR spectrum of BC were observed. The expansion in C–O–C etheric stretching vibrations seen around 1000–1100 cm^–1^ is due to surface carbohydrates in yeast groups. Similar to the BC structure, aliphatic C–H stretching vibrations were observed as a net peak around 2850 and 2930 cm^–1^, and C–C stretching vibrations were observed around 1553 and 1360 cm^–1^ for BC-yeast. Aliphatic C–H vibrations, on the other hand, prove aliphatic glucopyranose units at around 435–557 and 660 cm^–1^, similar to the pure BC structure. The peak intensity in the range of 3000–3600 cm^–1^, originating from the H-bond interactions between the surface –OH units in the BC structure, increased significantly due to the H-bonding of the –OH and −N–H groups originating from the binding of the yeast groups to the surface. However, the C = O stretching (amide I type) peak around 1650 cm-1 and the amide II peak around 1429 cm-1 of the BC-yeast originate from the protein groups of yeast attached to the BC. In addition, a C–H peak originating from aromatic units in the protein structures was observed at 825 cm^–1^ in the FTIR spectrum of the BC-yeast structure. All of these changes in the FTIR spectrum indicate that the yeast has been successfully immobilized to the BC.

The variation of the BC structures depending on the yeast cells was also confirmed by an X-ray spectroscopy technique. X-ray spectra of BC and BC-yeast samples are given in Figure 3. X-ray spectrum of the BC structure showed two distinct peaks at 14.56° and 22.60° 2-theta values. These net values showed us that the crystallinity ratio in the structure is high.^23^ The % crystallinity values were calculated for cellulose samples according to the formula^24^

$$C=100\times \frac{I_{200}{-I}_{non-cr}}{I_{200}}\left[\%\right]$$

In this crystallinity formula, *I*_200_ is the intensity of the peak of the Miller index called 200. In the calculation made according to this formulation, the crystallinity value for the relevant sample was approximately 85%. Despite the apparent cellulose peaks at 14.54° and 22.68°, 2-theta values after yeast immobilization in the relevant sample, a decrease in peak intensities, and an increase in amorphous character were observed. The % crystallinity value decreased to 78%. This decrease is due to the dense yeast groups bound to the surface. Yeast groups penetrated between the cellulose fibers and significantly disrupted the crystalline structure. This decrease in crystallinity proves to us that the obtained cellulose structure is quite suitable for yeast immobilization.

**Figure 3:**
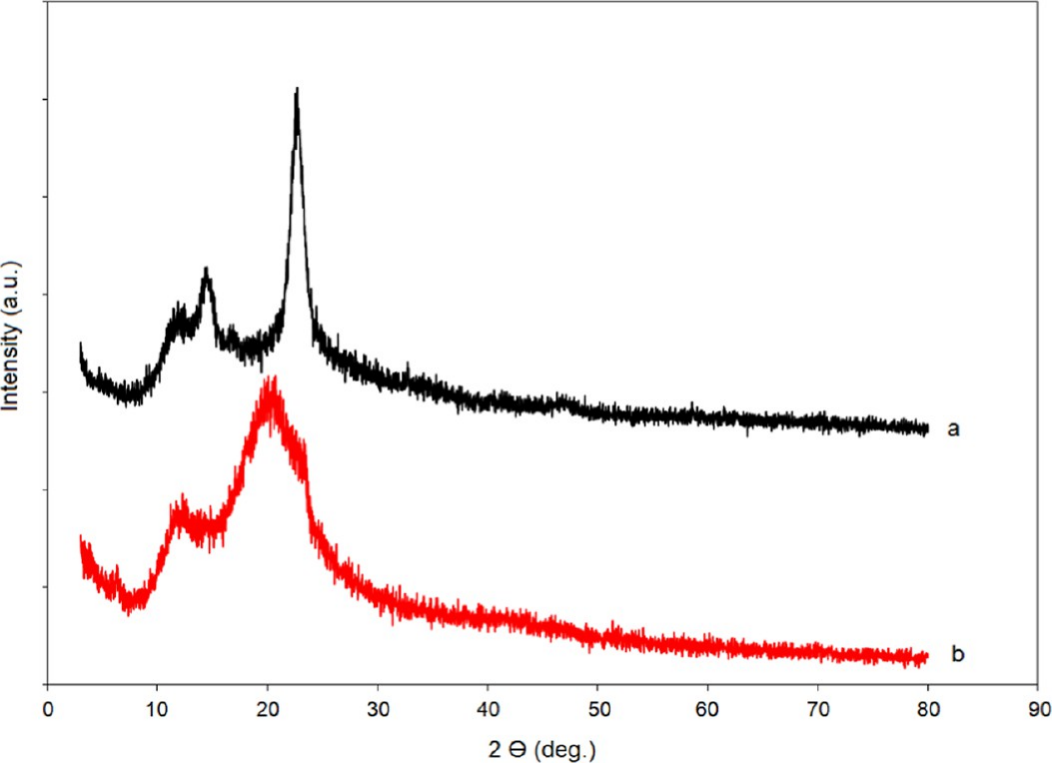
X-ray spectra of BC (a) and BC-yeast (b) samples.

### 3.2 Morphological Characterization of BC and BC-Yeast

Depending on the yeast immobilization, the changes in the cellulose surfaces and the surface morphology were examined in detail by SEM, AFM, and liquid contact angle measurements.

The SEM images of BC are given in Figure 4. When this figure was examined, a fibrous cellulose morphology was seen in general. Although this fibrous structure shows a layered film structure in places, the majority of the structure consists of thin fibers. The size distribution of the fibers was mostly around 60–200 nm. However, there were also fiber clusters with a size of 1–3 μm in places. The structure has a highly porous and void morphology and gives a 3D topography. Therefore, there is an ideal surface design for yeast immobilization.

**Figure 4:**
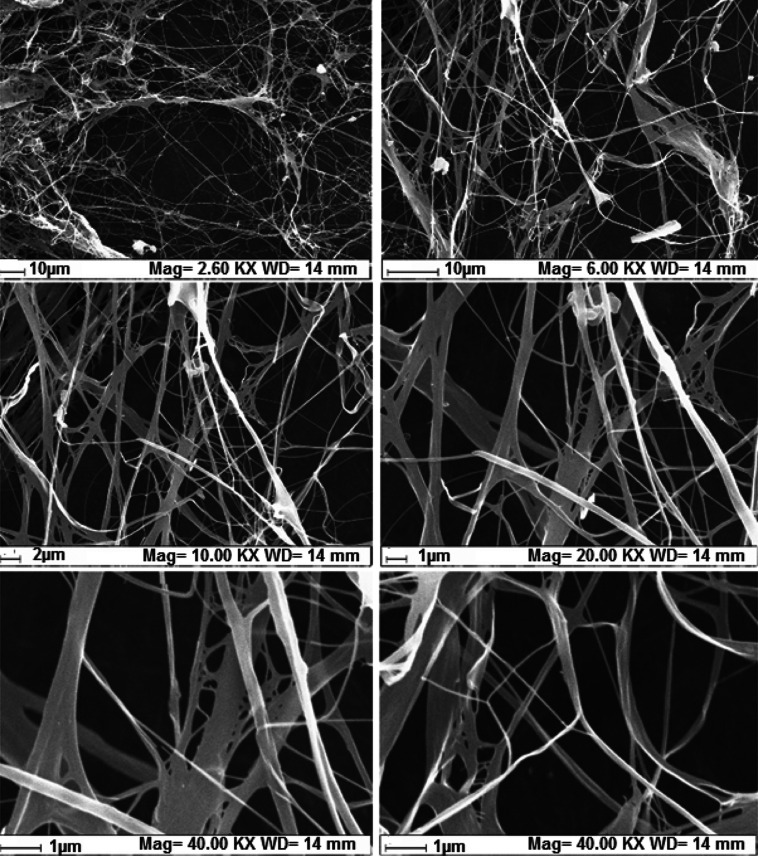
SEM images of BC sample at different magnifications (for all figures EHT = 20.00 kV, signal A = SE1).

The fiber diameter and size distribution of the BC structures are shown in Figure 5. It is seen that the fiber ring gives a uniform Gaussian distribution. The average fiber size distribution is around 144 nm. However, in some areas, broadband appearances have been created by placing these fibers side by side. The widest band formation is around 3100 nm. The resulting dense fiber structure, smooth BC band planes, and cellulose surface −OH structure create a suitable surface for yeast structures to adhere to the surface.

**Figure 5:**
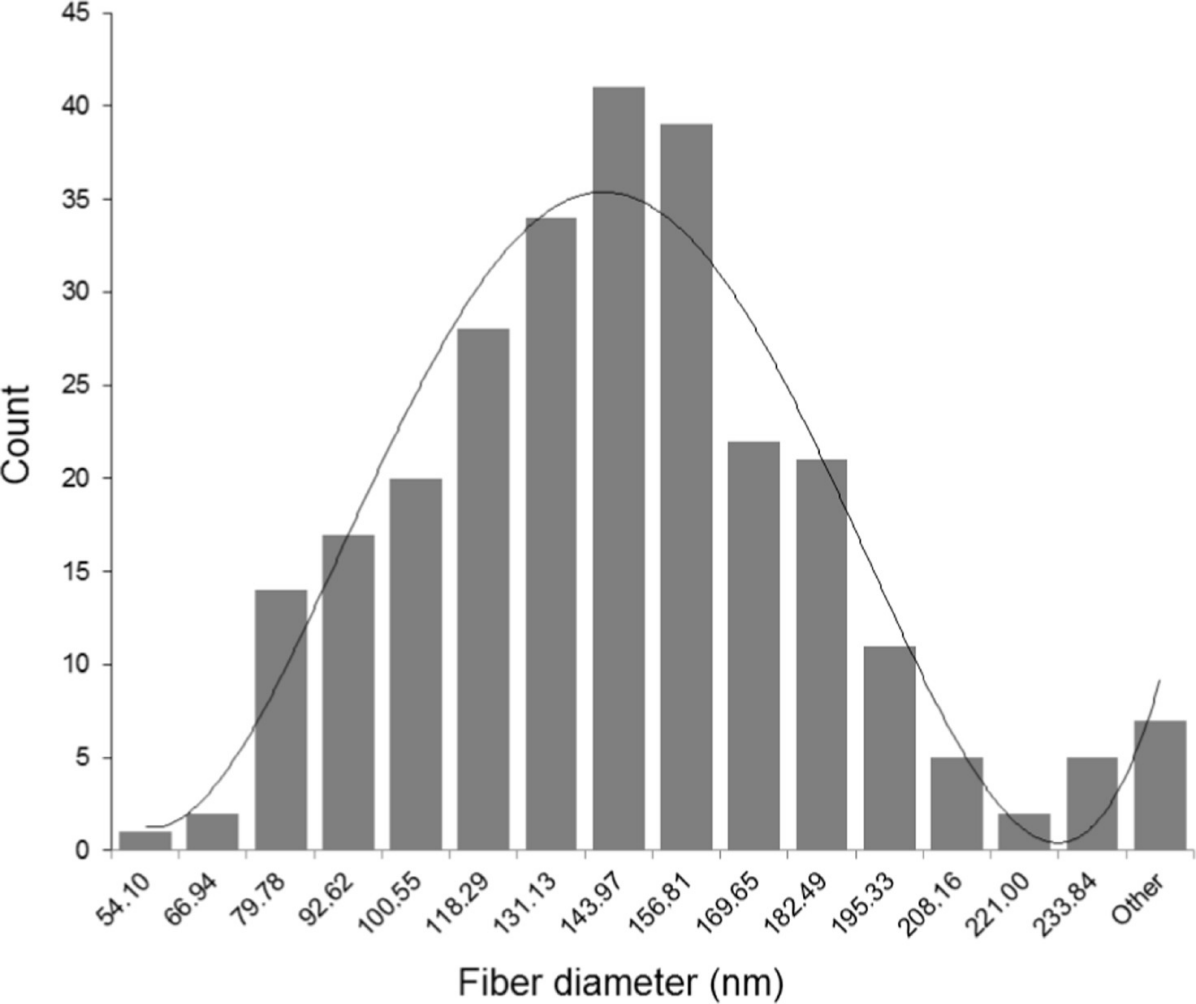
Fiber diameter distribution plot of BC.

Figure 6 shows the SEM images of BC-yeast obtained at different magnifications. It is clearly seen, especially at low magnifications, that yeast is homogeneously distributed over the entire surface. Yeast has grown in the form of spherical colonies, especially clinging to cellulose fibers. The growth took place in 3D and layers stacked on top of each other. When viewed at high magnifications, the agglomeration on the colonies is even more clearly seen, and the yeast structures are clearly seen as located between the fibers and on the fibers. It can be clearly stated that the cellulose-based fibrous structure obtained from these SEM images is an ideal structure for the immobilization of the yeast cells. Even at very high magnifications, it can be clearly demonstrated that the yeast structures fused to each other and moved toward a combined smooth film structure.

**Figure 6:**
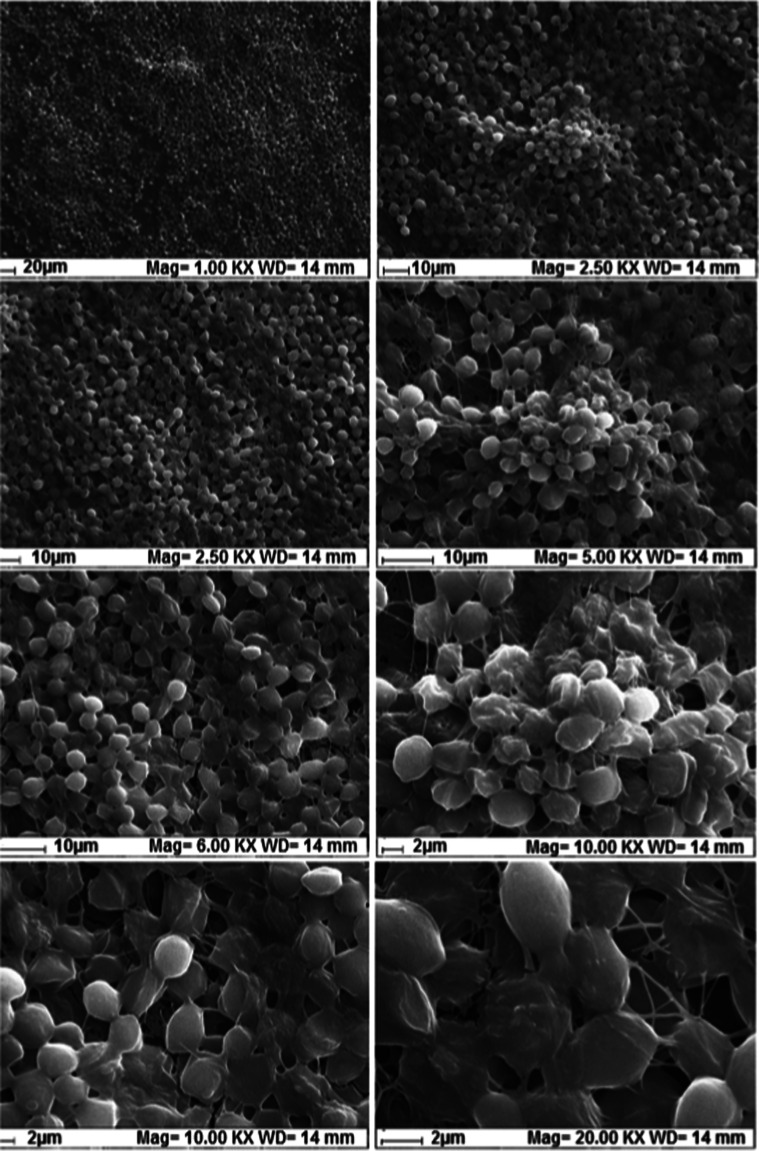
SEM images of BC-yeast samples (for all figures EHT = 20.00 kV, signal A = SE1).

AFM measurements were also made to reveal the presence of yeast in more detail, and are given in Figure 7. In these AFM measurements, images of BC and BC-yeast structures at three different magnifications are clearly seen. The smooth and natural fiber structure was revealed especially in BC structures. The fiber lines and gaps between the fibers are clearly evident (Figure 8). When the yeast cells adhered to the surface, this regular structure on the cellulose structure was disrupted, and yeast structures in the form of small mounds were clearly seen between the smoother layers. This also reveals the existence of yeast structures on BC.

**Figure 7:**
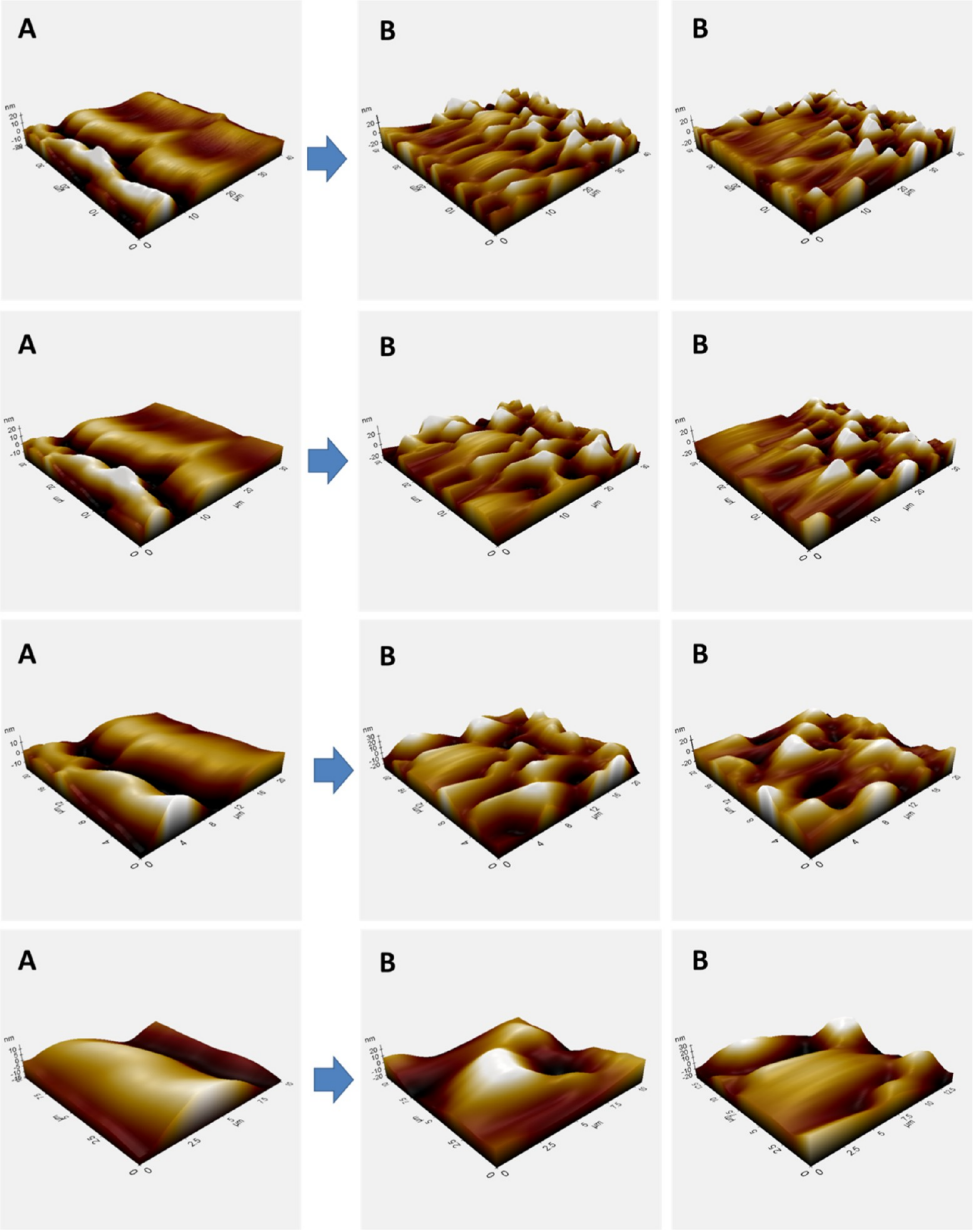
AFM images of BC (A) and BC-yeast (B) samples.

**Figure 8:**
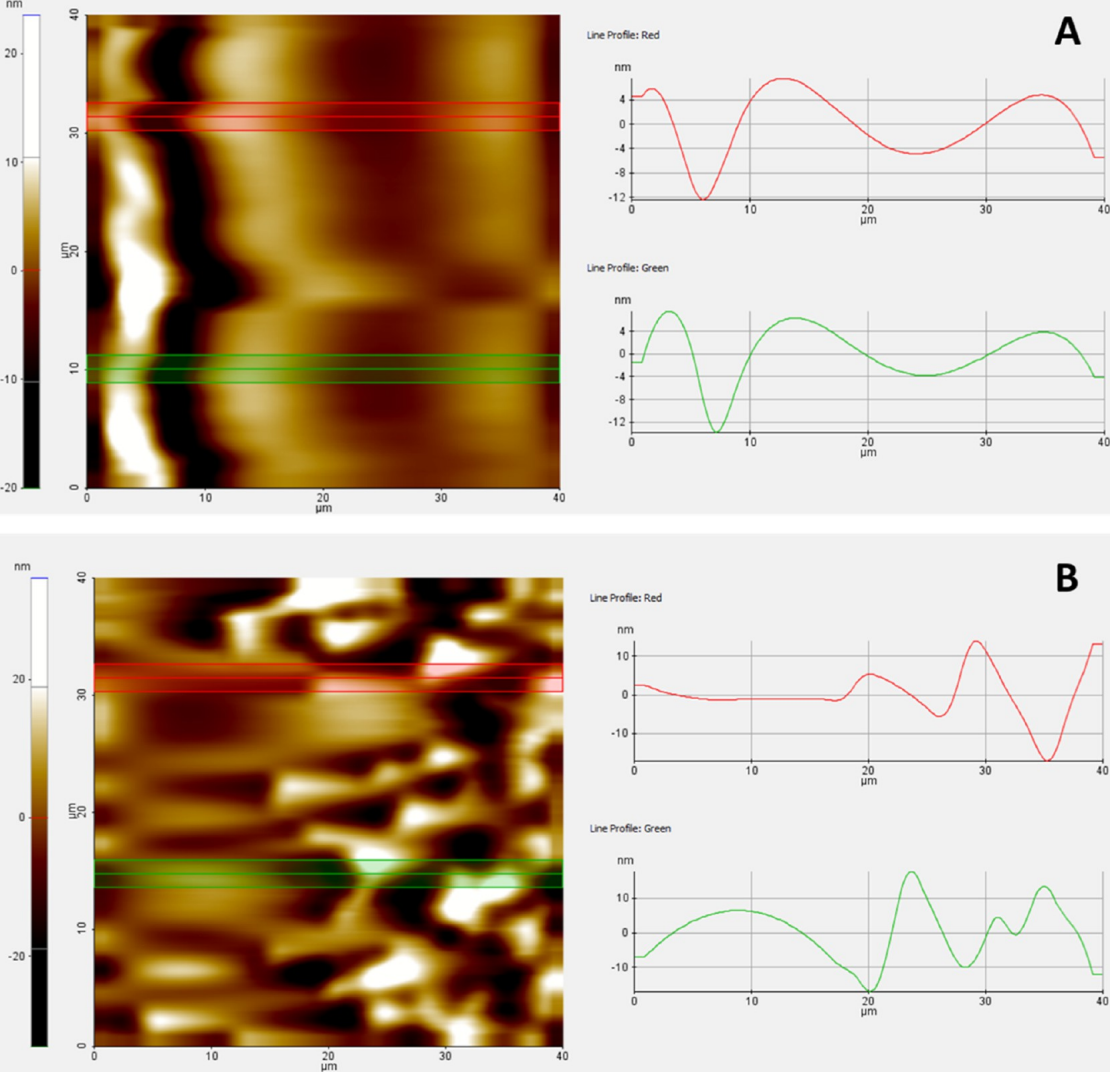
2D AFM images and surface roughness measurement of BC (A) and BC-yeast (B) samples.

The hydrophilicity of the structure by adhesion of the yeasts to the BC surface also changes. This change in hydrophilicity was determined, and fluid contact angle measurements were performed. According to these liquid contact angle measurements, the liquid contact angle of the surface of the BC revealed a value of approximately 41.49° due to the high surface hydrophilicity of the BC molecules, the cavity fiber structure obtained and the –OH groups on the surface (Figure 9a). When yeasts were attached to this surface, an increase in the liquid contact angle value was detected due to the phospholipid layers and hydrophobic groups around the yeast cells. The liquid contact angle value increased to approximately 63° (Figure 9b). These rising results prove to us that the targeted structure has been achieved. As a result, the cellulose structure of BC and immobilization of the presence of the yeast cells on these structures were successfully indicated by both chemical and surface analysis techniques.

**Figure 9:**
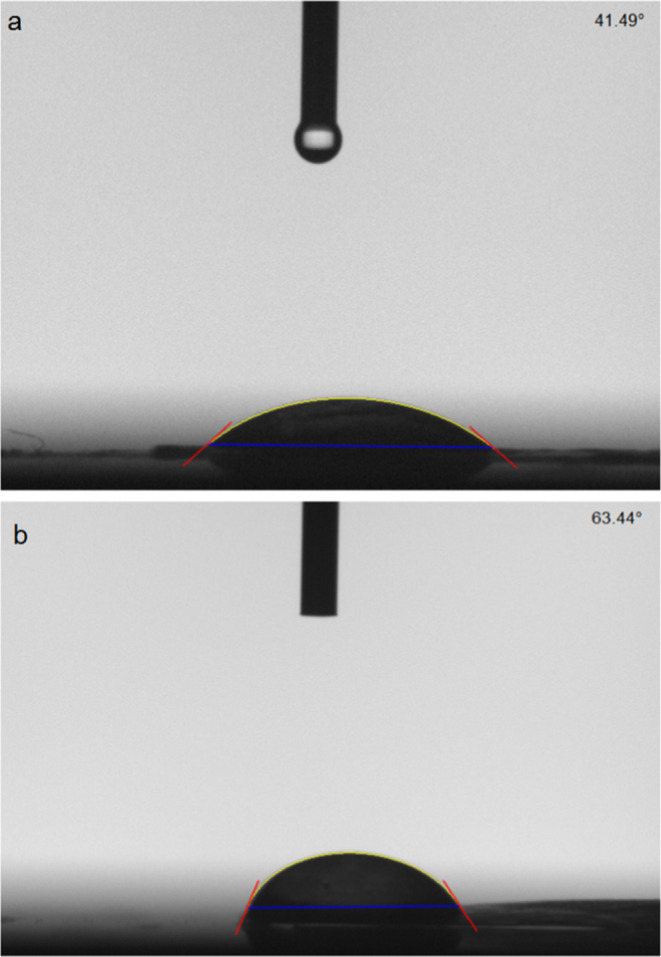
Liquid contact angles of BC (a) and BC-yeast (b) at different magnifications.

### 3.3 Yeast Immobilization Kinetics to BC Structure

In order to determine the optimum incubation time for the immobilization of yeast on BC, the yeasts were incubated in medium containing BC for 24, 48, 72, and 96 h. At the end of the relevant periods, samples were taken, and the yeast colonies attached to the fiber structure were counted. In this way, the number of colonies in a unit area was determined.

SEM images of yeasts attached to the BC structure at different incubation times are given in Figure 10. It was observed that the number of yeasts attached to the BC structure increased rapidly at first but then decreased slowly. In the attachment images obtained after 24 h, it was observed that the yeast colonies generally adhered in a single row. At the end of 48 h, it was observed that yeast attachments were multilayered and colony aggregation. However, SEM images at the end of 72 and 96 h showed large gaps between colonies. The reason for this can be associated with the level of glucose in the medium. As the yeast colonies attach, they quickly consume the glucose, and then the adhesion decreases. Figure 11 shows the number of yeast colonies adhering to the surface per unit area. When this figure was examined, the optimum incubation time for yeast attachment to the BC fiber structure was determined as 48 h.

**Figure 10:**
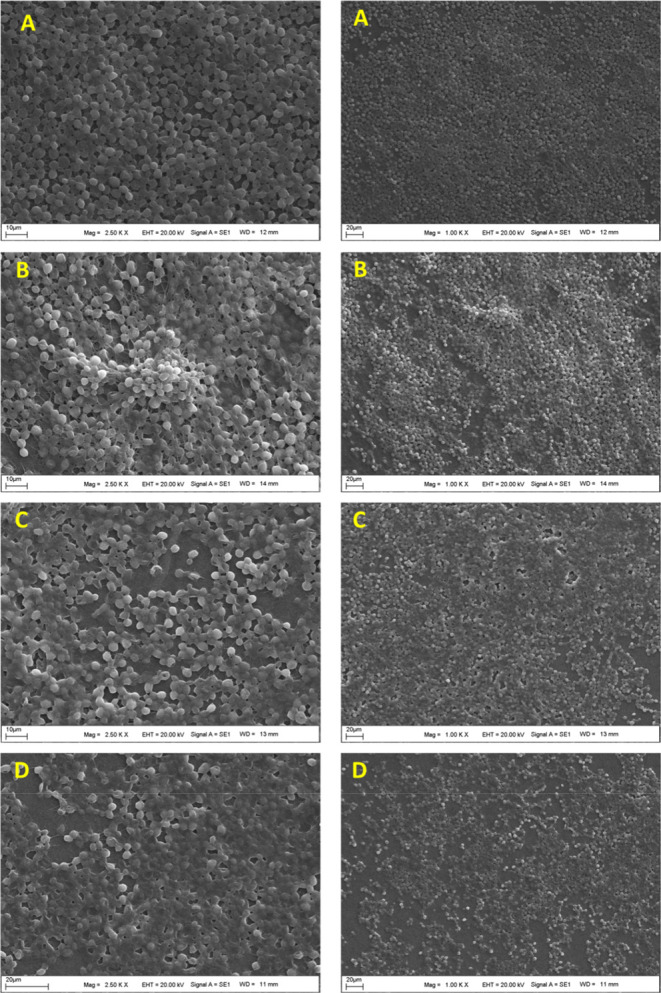
SEM images of BC-yeast surfaces after incubation for 24 (A), 48 (B), 72 (C), and 96 (D) h.

**Figure 11:**
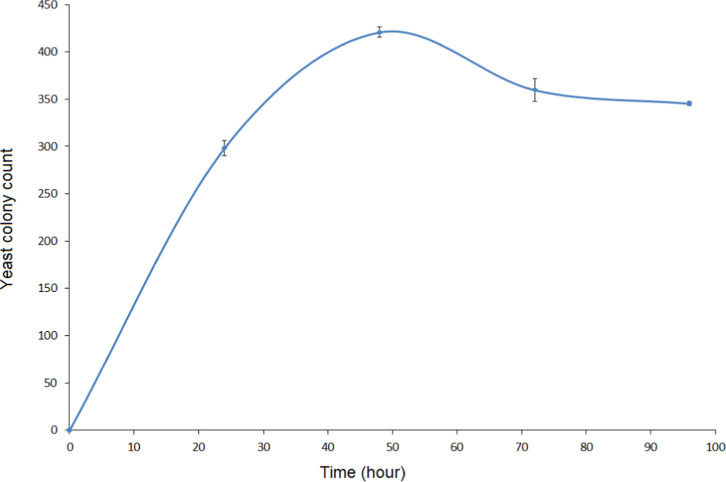
Number of yeast cell immobilized to the surface per unit area (1 μm × 1 μm) time graph.

### 3.4 Dye Removal by BC-Yeast

Textile dyes are one of the main pollutants used in many industrial areas. Most of these dyes are resistant to degradation and highly toxic.^25^ When these dark dyes are released into the environment without being sufficiently decolorized, they negatively affect the photosynthetic activity and dissolved oxygen level in the aquatic environment. Hence, it has serious negative effects on both the environment and organisms.^26–28^ Conventional treatment methods are insufficient to degrade these dyes, and the degradation of dyes by microorganisms and their enzyme systems is a remarkable and effective method for researchers.^29,30^

In this study, first, BC membranes were purified, and then wet and lyophilized BC samples were prepared. Then, yeast was immobilized on these BC samples, and the obtained BC-yeast sheets were used for dye removal. For dye removal studies, first, the BC-yeast sheets were cut into 0.5 cm pieces, and different amounts (3, 6, and 12) of BC-yeast samples were placed into 12 well plates containing 3 mL of dye solution. These were incubated at 30 °C under static and shaking (150 rpm) conditions for 24 h. As shown in Figure 12, dye removal percentages were 25%, 45%, and 52% for 3, 6, and 12 pieces of lyophilized BC-yeast sheets at static condition after 24 h, respectively. Under agitated conditions, no dye removal was observed for 3 pieces of lyophilized BC-yeast sheets, whereas 13% and 32% dye removal was obtained for 6 and 12 pieces of BC-yeast, respectively. When wet BC-yeast sheets were used, 28%, 40%, and 49% dye removal was determined under static conditions for 3, 6, and 12 pieces of BC-yeast, while the removal values recorded were 21%, 40%, and 50% under agitated conditions (Figure 13).

**Figure 12:**
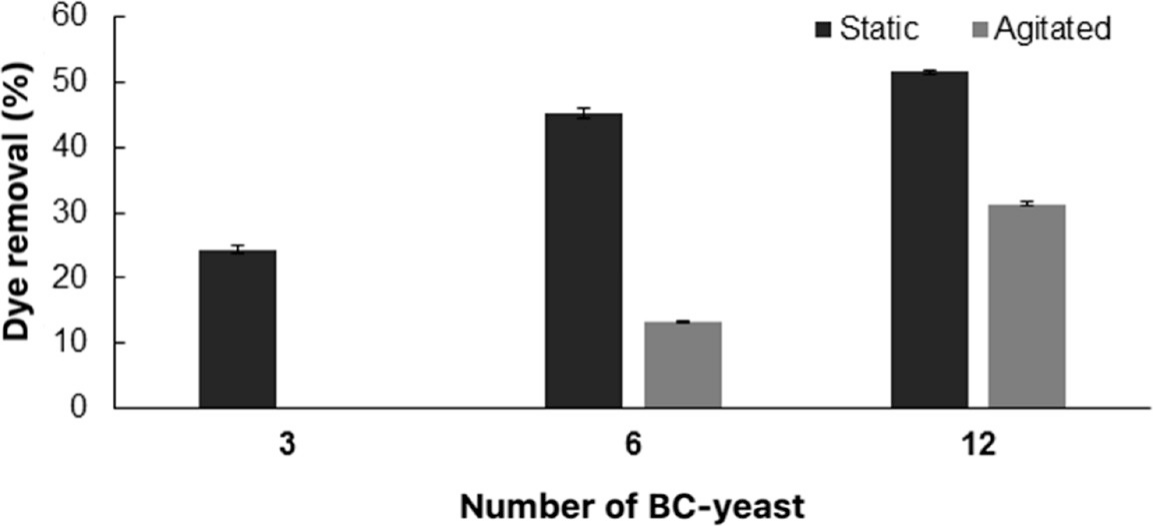
Removal of RB 171 by different amounts of lyophilized BC-yeast pieces (3, 6, and 12 pieces) under static and agitation conditions.

**Figure 13:**
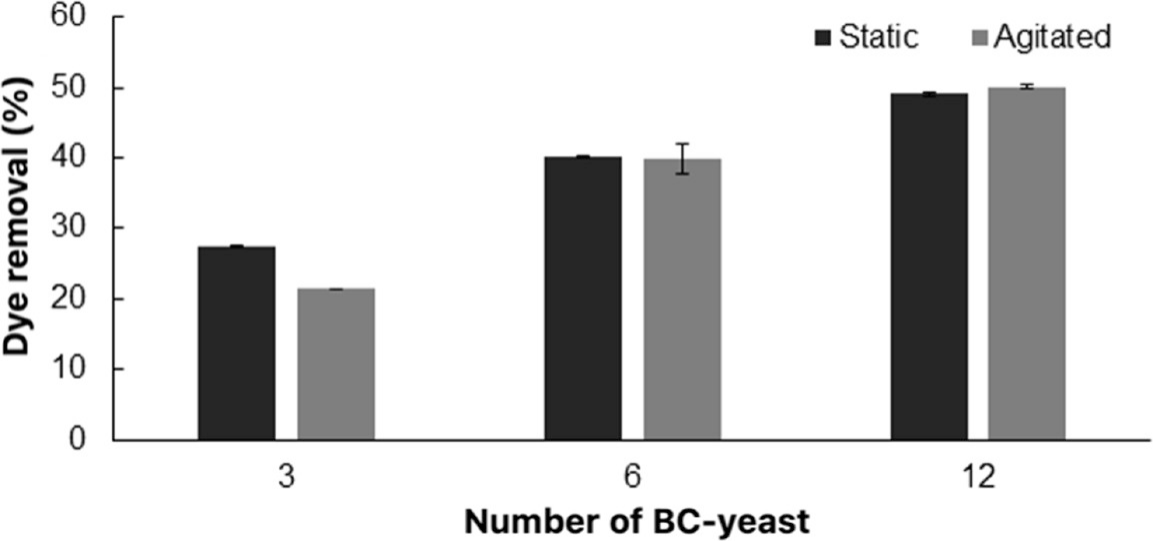
Removal of RB 171 by different amounts of wet BC-yeast pieces (3, 6, and 12 pieces) under static and agitation conditions.

Macroscopic images of RB 171 dye-containing solutions before and after treatment with 3, 6, and 12 pieces of lyophilized and wet BC-yeasts for 24 h under static and shaking conditions are given in Figure 14.

**Figure 14:**
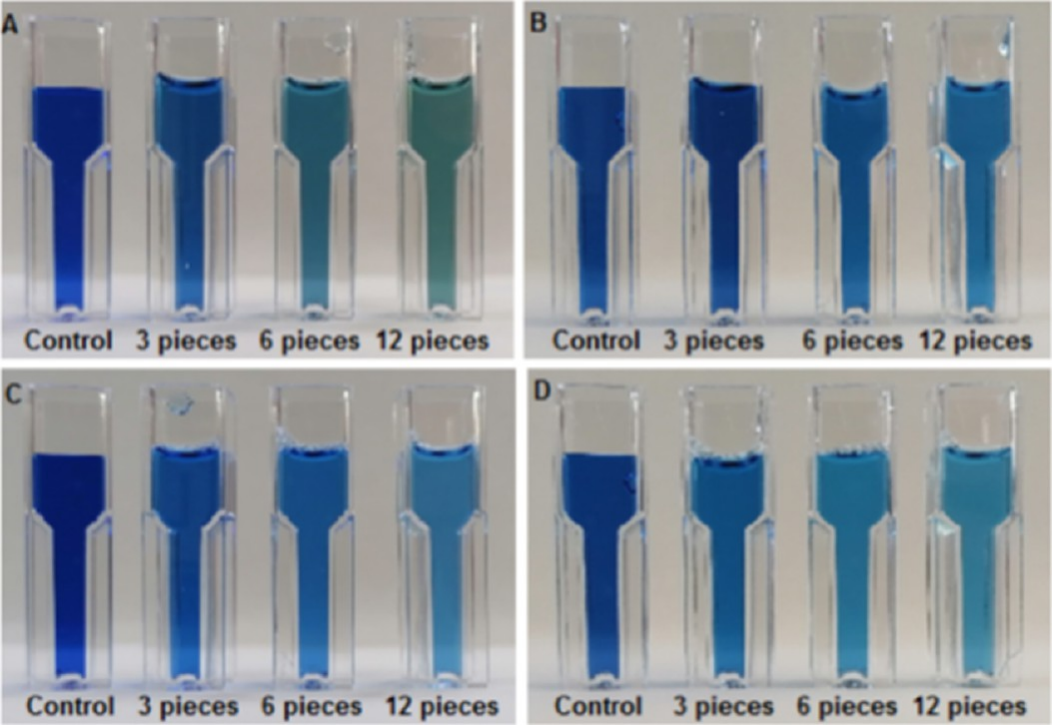
Macroscopic images of RB 171 dye-containing solutions (A- lyophilized static, B- lyophilized agitated, C- wet static, and D- wet agitated).

As a result, BC-yeast sheets were successfully used for dye removal. The immobilization of yeast on various immobilization matrices is very important for many industrial applications. Żywicka et al. (2016) immobilized *S. cerevisiae* on BC membranes obtained by three different strains of *G. xylinus* and they determined that the wet BC membrane obtained after 3 days of incubation was the most suitable carrier for the immobilization of yeast cells.^31^ In another study, the physicochemical properties and potential of the BC produced by *K. xylinus* as an immobilization matrix for *S. cerevisiae* and *Yarrowia lipolytica* were investigated under static conditions.^32^

### 3.5 DFT Calculation of Dye Adsorption onto BC-Yeast Sheets

In the study, DFT calculations were used to determine the intermolecular interactions involved in the adsorption of RB 171 onto the BC structure.^33^ These analyses were investigated to analyze the Frontier molecular orbitals, HOMO and LUMO, and the obtained HOMO, LUMO, and electronic parameters are given in Table 1.

**Table 1:** Calculated Electronic Parameters of RB 171, BC, and the Complex (BC + RB 171) Structures

	**E**_**HOMO**_ (**eV**)	**E**_**LUMO**_ (**eV**)	**Eg** (**eV**)	**χ** (**eV**)	**η** (**eV**)
**BC**	–6,99	1,32	8,31	2,84	4,1
**RB**-**171**	–5,20	–2,86	2,35	4,03	1,17
Complex	–5,39	–2,93	2,47	4,16	1,23

E_HOMO_ given in Table 1 for BC, RB 171, and complex structure indicates the electron-donating efficiency of a molecule and E_LUMO_ indicates the tendency of a molecule to accept electrons. The increase in the obtained E_HOMO_ value and the decrease in the energy gap confirmed a higher RB 171 adsorption on BC. On the other hand, the total energy and dipole moment were also determined to understand the effect of adsorption, stability, and reactivity of molecules. The higher value of the total energy indicated higher molecular stability. Dipole moment was directly related to the polarity of the molecule, which is related to the adsorption ability. The adsorption of RB 171 on BC increased due to the higher value of the dipole moment. Also, a higher χ value reduces the charge transfer ability of molecules and hence decreases the adsorption potential.^32^ The adsorption process also increases with a decrease of electrophilicity. Electronic parameters showed that cellulose has a high adsorption capacity.^22^ Also, the binding profile and solution phase energy values of the complex structure formed by the binding of BC, RB 171, and the complex are given in Figure 15 by theoretical calculations. From this figure, the energy of each species can be seen. The negative Eads value seen in this figure shows that the energy of the complex (BC + RB 171) is lower than the total energy of RB 171 and BC surfaces separately. This means that the adsorption of the ligand to the surface is energetically preferred, and this process is exothermic. In other words, energy is released when the ligand is bound to the surface. This energy value of approximately −24.48 kcal/mol (or −102.4 kJ/mol) indicates a very important and strong interaction for adsorption. This value is stronger than typical physical adsorption (weak van der Waals forces, usually <10–15 kcal/mol). An energy of this magnitude may indicate the formation of a strong hydrogen bond network or even an interaction strong enough to be considered close to (or within) chemical adsorption (chemisorption). There are probably multiple and/or specific interactions, such as strong hydrogen bonds between RB 171 and the BC surface. As a result, it was shown that under the modeled conditions, the RB 171 structure is energetically very strong and favorably bound to the BC-yeast surface. The adsorption process is exothermic. Therefore, it can be concluded that the BC-yeast structure can be effective in removing dye from aqueous solutions, and this result is in line with the results of experimental studies.

**Figure 15:**
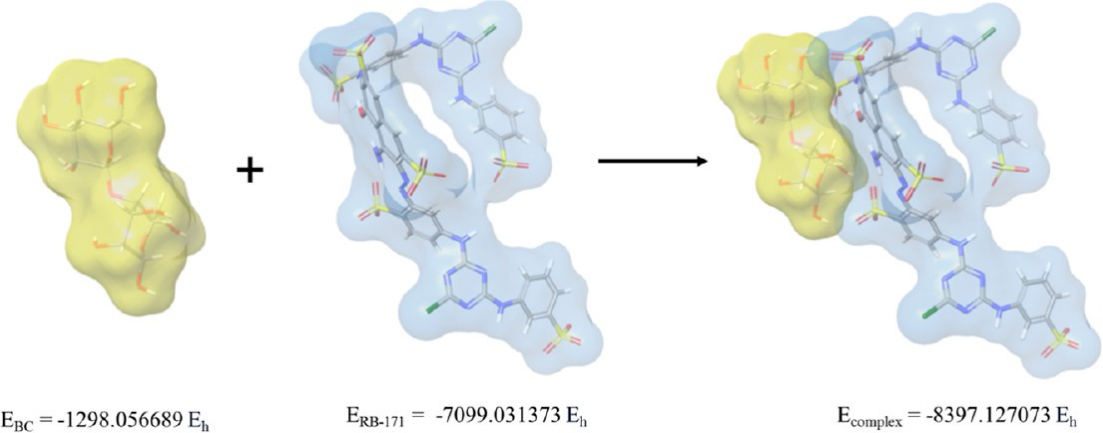
Theoretical binding profile and solution phase energy values of the complex structure formed by the binding of BC and RB 171.

## 4 Conclusion

In this study, *G. xylinus* B 759 could produce BC in a DAEM. Then, pure forms of wet and lyophilized BC sheets were obtained, and *S. cerevisiae* was successfully immobilized on these BC sheets. In addition, yeast immobilization was tested on lyophilized BC samples, and successful results were obtained. The optimum incubation time for yeast immobilization onto BC was 48 h. The BC-yeast could be used to remove the textile dye. Additionally, the adsorption process between BC-yeast and RB 171 was investigated using DFT calculations. According to these calculations, it was observed that the adsorption process was exothermic and there was a strong interaction between BC-yeast and RB 171. The results of all analyses proved the desired BC-yeast structure and showed that the BC-yeast sheets could also be used for dye removal.

## Data Availability

There is no legal/ethical problem in publishing the data.
